# Improved approximation of spatial light distribution

**DOI:** 10.1371/journal.pone.0176252

**Published:** 2017-04-28

**Authors:** David Kaljun, Tina Novak, Janez Žerovnik

**Affiliations:** 1 Faculty of Mechanical Engineering, University of Ljubljana, 1000 Ljubljana, Slovenia; 2 Institute of Mathematics, Physics and Mechanics, 1000 Ljubljana, Slovenia; IUMPA - Universitat Politecnica de Valencia, SPAIN

## Abstract

The rapid worldwide evolution of LEDs as light sources has brought new challenges, which means that new methods are needed and new algorithms have to be developed. Since the majority of LED luminaries are of the multi-source type, established methods for the design of light engines cannot be used in the design of LED light engines. This is because in the latter case what is involved is not just the design of a good reflector or projector lens, but the design of several lenses which have to work together in order to achieve satisfactory results. Since lenses can also be bought off the shelf from several manufacturers, it should be possible to combine together different off the shelf lenses in order to design a good light engine. However, with so many different lenses to choose from, it is almost impossible to find an optimal combination by hand, which means that some optimization algorithms need to be applied. In order for them to work properly, it is first necessary to describe the input data (i.e. spatial light distribution) in a functional form using as few as possible parameters. In this paper the focus is on the approximation of the input data, and the implementation of the well-known mathematical procedure for the separation of linear and nonlinear parameters, which can provide a substantial increase in performance.

## Introduction

The rapid worldwide evolution of the LED (Light emitting diode) industry has resulted in the implementation of LED elements in all kind of luminaries. Their technology means that energy consumption is much reduced, while at the same time there are endless possibilities of light engine design. In the case of LEDs lighting systems it is possible to deliver the light to the environment in a controlled way, although this leads to new problems such as finding the optimal lens or lens combination, the optimal LED to use, the optimal number of LEDs and the optimal rotation of each lens. The key to discovering a successful design process is the choice of the secondary optics. Currently there are more or less just two options for designing light engines. The first option is to have the know-how and the resources to design a specific lens in order to accomplish the task. However, the cost of resources coupled with the development and production of optical elements can be enormous. For this reason a lot of manufactures now make use of the second option, which is to use ready-made off the shelf lenses. These lenses are manufactured by a number of specialized companies, which offer different types of lenses for all the major LED brands. The trick here is to choose the best combination of lenses in order to get the most efficient system. The developer frequently makes use of a trial and error method, first choosing a good combination of lenses, and then simulating the system via Monte Carlo ray-tracing methods. The success of such procedures heavily depends on the engineer’s intuition and experience, sizeable computation resources are also needed to check the proposed design by means of simulations. However, analytical models and optimization tools could be used to speed up the design process, as well as to possibly improve the quality of solutions. An analytical model which could be used to describe the far field radiation pattern of a LED was recently proposed in [1, 2]. Later it was observed that the model could also be applied to LEDs with attached secondary optics [3]. Several optimization algorithms were designed and tested, showing that the model is accurate and provides an improvement in the field [4, 5]. In particular, the metaheuristics, such as the multi-start local search algorithm and genetic algorithms, proved to be excellent pre-processing methods for Newton’s method [6].

The Luminous intensity pattern of LEDs is mathematically described in form Eq (1). T3his form is a linear combination of nonlinear functions ∑*a*_*j*_*φ*_*j*_(***α***; *θ*) (see Eqs (7 and 28). Thus, for the calculation of the RMS error (see Eq (3)) it is more efficient to use the procedure which involves reduction of the linear parameters, which is well known from [7]. Using this procedure it has been possible to find desired solutions in a fraction of the time needed if usual discrete optimization methods are used. In turn this means that we are no longer confined to a HPC (High Performance Computing) unit but can run the approximation on a single processor desktop unit.

## Materials and methods

### The model

It is well-known that the luminous intensity pattern of LEDs can be represented as a sum of cosine-power functions

$$\begin{array}{c} I\left(\theta ;a,b,c\right)=I_{\max}\sum_{j=1}^{n}a_{j}\cos^{c_{j}}\left(\theta -b_{j}\right) \end{array}$$ (1)

(see in [2, 6]), where *θ* is the polar angle, *K* is the number of functions to sum, and *a*_*j*_, *b*_*j*_ and *c*_*j*_ are the function coefficients. For brevity, coefficients are written as vectors ***a*** = (*a*_1_, *a*_2_,…,*a*_*n*_)^*T*^, ***b*** = (*b*_1_, *b*_2_,…,*b*_*n*_)^*T*^ and ***c*** = (*c*_1_, *c*_2_,…,*c*_*n*_)^*T*^. In the paper, after the separation of the parameters ***a***, ***b*** and ***c***, vector notation ***α*** = (*b*_1_, *b*_2_,…,*b*_*n*_, *c*_1_, *c*_2_,…,*c*_*n*_)^*T*^ is also used. The interval range of the coefficients is: *a*_*j*_ ∈ [0, 1], *b*_*j*_ ∈ [−90,90] and *c*_*j*_ ∈ [0, 100] for every *j* = 1,2,…,*n*. In [6], discrete optimization algorithms work on finite subsets where the possible values are

$$\begin{array}{ccc} a_{j} & \in & \{0,0.001,0.002,\ldots ,1\},\\ b_{j} & \in & \{-90,-89.9,-89.8,\ldots ,89.9,90\},\\ c_{j} & \in & \{0,1,2,...,100\}. \end{array}$$ (2)

For given data (*θ*_*i*_, *I*_*M*_(*θ*_*i*_)), *i* = 1,2,…,*m*, (*N* >> 39*n*) the optimal parameters minimizing the function

$$\begin{array}{c} RMS\left(a,b,c\right)=\sqrt{\frac{1}{N}\sum_{i=1}^{m}{\left(I_{M}\left(\theta_{i}\right)-I\left(\theta_{i};a,b,c\right)\right)}^{2}} \end{array}$$ (3)

are determined. In Eq (3), *N* is the number of measured points in the input data, *I*_*M*_(*θ*_*i*_) is the measured luminous intensity value at a polar angle *θ*_*i*_, and *I*(*θ*_*i*_, ***a***, ***b***, ***c***) the calculated luminous intensity value at the given polar angle *θ*, and the given triplet of vectors (***a***, ***b***, ***c***) from the finite discrete subset of [0, 1]^*n*^ × [−90, 90]^*n*^ × [0, 100]^*n*^. The *RMS* function represents the error of the approximation named *RMSp*, and is defined by the equation

$$\begin{array}{c} RMSb\left(a,b,c\right)=\frac{100\cdot m\cdot RMS(a,b,c)}{\sum_{i=1}^{m}I_{M}\left(\theta_{i}\right)}\left[\%\right]. \end{array}$$ (4)

In order to simplify the problem, it is sufficient to consider the standard least squares problem

$$\begin{array}{c} G\left(a,b,c\right)=\sum_{i=1}^{m}{\left(I_{M}\left(\theta_{i}\right)-I\left(\theta_{i};a,b,c\right)\right)}^{2}\;. \end{array}$$ (5)

The function *G* can be used since to minimize the *RMS* function (3), i.e. finding parameter values a*, b*, c* for which the value RMS s minimal, is equivalent to minimize the function *G*. Due to the form of the function (1), the problem (5) is one of the so-called “separable nonlinear least squares problems”, which were studied already in [7, 8]. In the next subsection we present the procedure for reducing one third of the parameters in the nonlinear least square problems where the functions in the linear square problem have a special form.

### Nonlinear least square problems whose variables can be separated

#### Separation of the linear variables

Consider the real given data as

$$\begin{array}{c} (t_{i},y_{i})\;,\;i=1,\ldots ,m. \end{array}$$ (6)

Denote by ***a*** and ***α*** two independent vectors

$$\begin{array}{c} a={\left(a_{1},\ldots ,a_{n}\right)}^{T}\in R^{n}\;\text{and}\;\alpha ={\left(\alpha_{1},\ldots ,\alpha_{k}\right)}^{T}\in R^{k} \end{array}$$

and let

$$\begin{array}{c} \eta \left(a,\alpha ;t\right)=\sum_{j=1}^{n}a_{j}\varphi_{j}\left(\alpha ;t\right) \end{array}$$ (7)

be nonlinear models, where *φ*_*j*_ are functions, continuously differentiable with respect to ***α***, and *t* is a real variable. If instead of *t* a vector ***t*** = (*t*_1_,…,*t*_*m*_)*^T^* is taken, one should write

$$\begin{array}{c} \eta \left(a,\alpha \right)={\left(\eta \left(a,\alpha ;t_{1}\right),\ldots ,\eta \left(a,\alpha ;t_{m}\right)\right)}^{T} \end{array}$$

and

$$\begin{array}{c} \varphi_{j}\left(\alpha \right)={\left(\varphi_{j}\left(\alpha ;t_{1}\right),\varphi_{j}\left(\alpha ;t_{2}\right),\ldots ,\varphi \left(\alpha ;t_{m}\right)\right)}^{T}. \end{array}$$

We also write ***y*** = (*y*_1_,…,*y*_*m*_)^*T*^ for the given values in Eq (6).

**Problem**: Find the values of parameters ***a*** and ***α*** that minimize the nonlinear functional

$$\begin{array}{ccc} r\left(a,\alpha \right)=||y-{\eta \left(a,\alpha \right)||}^{2} & = & \sum_{i=1}^{m}{\left(y_{i}-\eta \left(a,\alpha ;t_{i}\right)\right)}^{2}= \end{array}$$ (8)

$$\begin{array}{ccc} & = & \sum_{i=1}^{m}{\left(y_{i}-\sum_{j=1}^{n}a_{j}\varphi_{j}\left(\alpha ;t_{i}\right)\right)}^{2}\;. \end{array}$$ (9)

Let Φ(***α***) be the matrix function

$$\begin{array}{c} \Phi \left(\alpha \right)=\left[\varphi_{1}\left(\alpha \right),\varphi_{2}\left(\alpha \right),\ldots ,\varphi_{n}\left(\alpha \right)\right]=\left[\begin{array}{cccc} \varphi_{1}\left(\alpha ;t_{1}\right) & \varphi_{2}\left(\alpha ;t_{1}\right) & \ldots & \varphi_{n}\left(\alpha :t_{1}\right)\\ \varphi_{1}\left(\alpha ;t_{2}\right) & \varphi_{2}\left(\alpha ;t_{2}\right) & \ldots & \varphi_{n}\left(\alpha :t_{2}\right)\\ \vdots & \vdots & \ddots & \vdots \\ \varphi_{1}\left(\alpha ;t_{m}\right) & \varphi_{2}\left(\alpha ;t_{m}\right) & \ldots & \varphi_{n}\left(\alpha :t_{m}\right) \end{array}\right]. \end{array}$$ (10)

The sum $\sum_{j=1}^{n}a_{j}\varphi_{j}\left(\alpha ;t_{i}\right)$ (see Eq (9)) is the *i*-th component of the vector

$$\begin{array}{c} a_{1}\varphi_{1}\left(\alpha \right)+a_{2}\varphi_{2}\left(\alpha \right)+\ldots +a_{n}\varphi_{n}\left(\alpha \right)\;=\;\left[\begin{array}{c} a_{1}\varphi_{1}\left(\alpha ;t_{1}\right)+a_{2}\varphi_{2}\left(\alpha ;t_{1}\right)+\ldots +a_{n}\varphi_{n}\left(\alpha ;t_{1}\right)\\ a_{1}\varphi_{1}\left(\alpha ;t_{2}\right)+a_{2}\varphi_{2}\left(\alpha ;t_{2}\right)+\ldots +a_{n}\varphi_{n}\left(\alpha ;t_{2}\right)\\ \vdots \\ a_{1}\varphi_{1}\left(\alpha ;t_{m}\right)+a_{2}\varphi_{2}\left(\alpha ;t_{m}\right)+\ldots +a_{n}\varphi_{n}\left(\alpha ;t_{m}\right) \end{array}\right]. \end{array}$$ (11)

Therefore, the functional *r* is geometrically the length of the vector

$$\begin{array}{c} y-\left(a_{1}\varphi_{1}\left(\alpha \right)+a_{2}\varphi_{2}\left(\alpha \right)+\ldots +a_{n}\varphi_{n}\left(\alpha \right)\right)\;. \end{array}$$

For a given vector ***y*** the value of *r* is minimal if and only if the sum (vector) *a*_1_***φ***_1_(***α***) + *a*_2_***φ***_2_(***α***) + … + *a*_*n*_***φ***_*n*_(***α***) is the orthogonal projection of ***y*** onto the subspace $L\{\varphi_{1}(\alpha ),\varphi_{2}(\alpha ),\ldots ,\varphi_{n}(\alpha )\}$. For each ***α***, the linear operator

$$\begin{array}{c} P_{\Phi (\alpha )}=\Phi \left(\alpha \right)\Phi^{+}\left(\alpha \right) \end{array}$$

is the orthogonal projection on the linear space spanned by the columns of the matrix Φ(***α***) (see Remark 2), i.e. the linear space $L\{\varphi_{1}(\alpha ),\varphi_{2}(\alpha ),\ldots ,\varphi_{n}(\alpha )\}$.

**Remark 1** *The matrix* Φ^+^(***α***) *is the generalized inverse or so called Moore-Penrose pseudoinverse*. *For every m* × *n matrix A*, *there exists a unique n* × *m matrix X*, *such that*

$$\begin{array}{ccc} AXA & = & A \end{array}$$ (12)

$$\begin{array}{ccc} XAX & = & X \end{array}$$ (13)

$$\begin{array}{ccc} {\left(AX\right)}^{⊤} & = & AX \end{array}$$ (14)

$$\begin{array}{ccc} {\left(XA\right)}^{⊤} & = & XA. \end{array}$$ (15)

*A*^+^ *is defined to be X*. *The proof can be found in* [9].

**Remark 2** *From the definition of the Moore-Penrose pseudoinverse we obtain*

$$\begin{array}{c} P_{\Phi (\alpha )}\Phi \left(\alpha \right)=\Phi \left(\alpha \right)\Phi^{+}\left(\alpha \right)\Phi \left(\alpha \right)=\Phi \left(\alpha \right) \end{array}$$

*and hence*

$$\begin{array}{c} P_{\Phi (\alpha )}\varphi_{j}\left(\alpha \right)=\varphi_{j}\left(\alpha \right) \end{array}$$

*for every j* = 1,…,*n*. *Consider that v is a nonzero vector in the orthogonal complement of the subspace* $L\{\varphi_{1}(\alpha ),\varphi_{2}(\alpha ),\ldots ,\varphi_{n}(\alpha )\}$ *according to the usual scalar product*. *Such a vector v is characterized by the vanishing of the scalar product of the vectors v and **φ**_j_(**α**) for every j* = 1,…,*n*. *Therefore*,

$$\begin{array}{c} {\left(\Phi \left(\alpha \right)\right)}^{⊤}v=0\;. \end{array}$$

*Hence the following is true*

$$\begin{array}{ccc} {\left(\Phi^{+}\left(\alpha \right)\right)}^{⊤}{\left(\Phi \left(\alpha \right)\right)}^{⊤}v & = & 0\\ {\left(\Phi \left(\alpha \right)\Phi^{+}\left(\alpha \right)\right)}^{⊤}v & = & 0\\ \Phi \left(\alpha \right)\Phi^{+}\left(\alpha \right)v & = & 0\\ P_{\Phi (\alpha )}v & = & 0\;. \end{array}$$

Another linear operator needed here is

$$\begin{array}{c} P_{\Phi (\alpha )}^{⊥}=I-P_{\Phi (\alpha )}\;. \end{array}$$ (16)

Geometrically, this is the projection onto the orthogonal complement of the linear space spanned by the columns of the matrix Φ(***α***). For any given ***α*** we have

$$\begin{array}{c} r\left(\hat{a},\alpha \right)=\min_{a}r\left(a,\alpha \right)=||y-\Phi \left(\alpha \right)\Phi^{+}\left(\alpha \right){y||}^{2}=\left|\right|P_{\Phi (\alpha )}^{⊥}y{\left|\right|}^{2} \end{array}$$

and

$$\begin{array}{c} \hat{a}\left(\alpha \right)\equiv \Phi^{+}\left(\alpha \right)y\;. \end{array}$$ (17)

Define the modified functional as

$$\begin{array}{c} r_{2}\left(\alpha \right)=\left|\right|P_{\Phi (\alpha )}^{⊥}y{\left|\right|}^{2}. \end{array}$$ (18)

Once a critical point (minimizer) $\hat{\alpha }$ of *r*_2_ is found, $\hat{a}$ can be calculated using Eq (17) on $\hat{\alpha }$, i.e.

$$\begin{array}{c} \hat{a}=\Phi^{+}\left(\hat{\alpha }\right)y\;. \end{array}$$ (19)

**Theorem 3 (Golub, Pereyra 1973)** *Let r(**a***, ***α***) *and r_2_*(***α***) *be defined as above*. *We assume that in the open set* $\Omega \subset R^{k}$, *the matrix* Φ(***α***) *has a constant rank r* ≤ min(*m*, *n*).

1. *If* $\hat{\alpha }$ *is a critical point (or a global minimizer for* ***α*** ∈ Ω*) of r*_2_(***α***), *and*
  $$\begin{array}{c} \hat{a}=\Phi^{+}\left(\hat{\alpha }\right)y \end{array}$$ (20)
  *then* $\left(\hat{a},\hat{\alpha }\right)$ *is a critical point of r*(***a***, ***α***) (*or a global minimizer for* ***α*** ∈ *Ω*) *and* $r\left(\hat{a},\hat{\alpha }\right)=r_{2}\left(\hat{\alpha }\right)$.
2. *If* $\left(\hat{a},\hat{\alpha }\right)$ *is a global minimizer of r*(***a***, ***α***) *for* ***α*** ∈ Ω, *then* $\hat{\alpha }$ *is a global minimizer of* *r*_2_(***α***) *in* Ω *and* $r_{2}\left(\hat{\alpha }\right)=r\left(\hat{a},\hat{\alpha }\right)$. *Furthermore, if there is an unique* $\hat{a}$ *among the minimizing pairs of r*(***a***, ***α***), *then* $\hat{a}$ *must satisfy* Eq (20).

For the proof see [8], where the method of decimation of the functional *r*_2_ is given after the theorem. The orthogonal transformation of the matrix Φ into “trapezoidal” form is used. Since we use C++ in the computations, in the next paragraph we shall present the method of simplification of the functional *r*_2_ in SVD manner.

#### Computing in C++, Singular Value Decomposition

In the Armadillo library (a high quality linear algebra library (matrix maths) for the C++ language, which aims to provide the ease of use and performance of MATLAB functions in more low level programming. “http://arma.sourceforge.net/”) there is the command **svd** for calculating the Singular Value Decomposition. For every rectangular *m* × *n* matrix Φ, where *m* ≥ *n*, the command **svd** gives us the matrices *U* and *V* and the vector *s*. The matrices *U* and *V* are orthogonal matrices of dimension *m* × *m* and *n* × *n* respectively. Further, *s* is the vector of the singular values *s*_1_, *s*_2_,…,*s*_*n*_. Let *S* be the diagonal matrix *S* = diag(*s*_1_,…,*s*_*n*_). We can assume that

$$\begin{array}{c} s_{1}\geq s_{2}\geq \ldots \geq s_{n}\geq 0. \end{array}$$ (21)

If rank(Φ) = *r*, then *s*_*r*+1_ = … = *s*_*n*_ = 0. If rank(Φ) = rank(*S*) = *r*, then denote by *S*_*r*_ the invertible diagonal matrix

$$\begin{array}{c} S_{r}=\left[\begin{array}{cccc} s_{1} & 0 & \ldots & 0\\ 0 & s_{2} & \ldots & 0\\ \vdots & \vdots & \ddots & \vdots \\ 0 & 0 & \ldots & s_{r} \end{array}\right]. \end{array}$$

Denote by Σ the *m* × *n* matrix defined by

1. if *r* = *n*, we write $\Sigma =\left[\frac{S}{0}\right]$,
2. if *r* < *n*, then $\Sigma =\left[\frac{\begin{array}{cc} S_{r}\;| & 0 \end{array}}{\begin{array}{cc} 0\;| & 0 \end{array}}\right]$.

Thus,

$$\begin{array}{c} \Phi \left(\alpha \right)=U\left(\alpha \right)\Sigma \left(\alpha \right)V^{T}\left(\alpha \right) \end{array}$$ (22)

(see [10], the alternative representation of SVD). The Moore-Penrose pseudoinverse of the matrix Σ is the following *n* × *m* matrix

1. if *r* = *n*, then $\Sigma^{+}=\left[S^{-1}|0\right]$,
2. if *r* < *n*, then $\Sigma^{+}=\left[\frac{\begin{array}{cc} S_{r}^{-1}\;| & 0 \end{array}}{\begin{array}{cc} 0\;| & 0 \end{array}}\right]$.

Therefore, the Moore-Penrose pseudoinverse of the matrix Φ is the matrix

$$\begin{array}{c} \Phi^{+}\left(\alpha \right)=V\left(\alpha \right)\Sigma^{+}\left(\alpha \right)U^{T}\left(\alpha \right)\;. \end{array}$$ (23)

The more simple form of the matrix of the projection *P*_Φ_ can be easily calculated by

$$\begin{array}{ll} P_{\Phi \left(\alpha \right)} & =\Phi \left(\alpha \right)\Phi^{+}\left(\alpha \right)=U\left(\alpha \right)\Sigma \left(\alpha \right)V^{T}\left(\alpha \right)V\left(\alpha \right)\Sigma^{+}\left(\alpha \right)U^{T}\left(\alpha \right)=\\ & =U\left(\alpha \right)\Sigma \left(\alpha \right)\Sigma^{+}\left(\alpha \right)U^{+}\left(\alpha \right)=U\left(\alpha \right)\left[\frac{\begin{array}{cc} I_{r}\;| & 0 \end{array}}{\begin{array}{cc} 0\;| & 0 \end{array}}\right]U^{T}\left(\alpha \right), \end{array}$$

where *I*_*r*_ denote the identity matrix of the dimension *r* × *r* for *r* = 1,…,*n* − 1. The orthogonal projection $P_{\Phi (\alpha )}^{⊥}$ (see Eq (16)) has the form

$$P_{\Phi \left(\alpha \right)}^{⊥}=U\left(\alpha \right)\left[\frac{\begin{array}{cc} 0\;| & 0 \end{array}}{\begin{array}{cc} 0\;| & I_{m-r} \end{array}}\right]U^{T}\left(\alpha \right).$$

From the given vector ***y*** = (*y*_1_,…,*y*_*m*_)^*T*^ another vector $\tilde{y}\left(\alpha \right)$ can be defined by

$$\begin{array}{c} \tilde{y}\left(\alpha \right)\;=\;U^{T}\left(\alpha \right)y. \end{array}$$ (24)

The functional *r*_2_ (see Eq (18)) can be written as

$$\begin{array}{cc} r_{2}\left(\alpha \right) & =||U\left(\alpha \right)\left[\frac{\begin{array}{cc} 0\;| & 0 \end{array}}{\begin{array}{cc} 0\;| & I_{m-r} \end{array}}\right]U^{T}\left(\alpha \right)y|{|}^{2}=\\ & =||U\left(\alpha \right)\left[\frac{\begin{array}{cc} 0\;| & 0 \end{array}}{\begin{array}{cc} 0\;| & I_{m-r} \end{array}}\right]\tilde{y}\left(\alpha \right)|{|}^{2}. \end{array}$$

If we write the vector $\tilde{y}$ in a block form such as

$$\tilde{y}\left(\alpha \right)=\left[\frac{{\tilde{y}}_{\left[r\right]}\left(\alpha \right)}{{\tilde{y}}_{\left[m-r\right]}\left(\alpha \right)}\right]$$ (25)

and since the orthogonal transformation (in our case *U*(***α***)) is isometric, we obtain

$$r_{2}\left(\alpha \right)=||\left[\frac{\begin{array}{cc} 0\;| & 0 \end{array}}{\begin{array}{cc} 0\;| & I_{m-r} \end{array}}\right]\tilde{y}\left(\alpha \right)|{|}^{2}=||{\tilde{y}}_{\left[m-r\right]}\left(\alpha \right)|{|}^{2}.$$ (26)

#### Model $\sum_{j=1}^{3}a_{j}\;{\text{cos}}^{c_{j}}\left(\theta -b_{j}\right)$

Let

$$\begin{array}{c} a={\left(a_{1},a_{2},a_{3}\right)}^{T}\in R^{3}\;,\;\alpha ={\left(b_{1},b_{2},b_{3},c_{1},c_{2},c_{3}\right)}^{T}\in R^{6}\;\text{and}\;\beta ={\left(b,c\right)}^{T}\in R^{2} \end{array}$$

be independent vectors. Denote by pr_*j*_ the projection

$$\begin{array}{ccc} {\text{pr}}_{j}:R^{6} & ⟼ & R^{2}\\ {\left(b_{1},b_{2},b_{3},c_{1},c_{2},c_{3}\right)}^{T} & ⟼ & {\left(b_{j},c_{j}\right)}^{T} \end{array}$$

for *j* = 1, 2, 3. Let *ψ* be the function, defined by

$$\begin{array}{ccc} \psi :R^{2}\times R & ⟶ & R\\ \left(\beta ;\theta \right) & ⟼ & \cos^{c}\left(\theta -b\right)\;. \end{array}$$ (27)

To write the function $\sum_{j=1}^{3}a_{j}\cos{\left(\theta -b_{j}\right)}^{c_{j}}$ in the form Eq (7), we take

$$\begin{array}{ccc} \varphi_{1}\left(\alpha ;\theta_{i}\right) & = & \psi ({\text{pr}}_{1}\left(\alpha \right);\theta_{i})\\ \varphi_{2}\left(\alpha ;\theta_{i}\right) & = & \psi ({\text{pr}}_{2}\left(\alpha \right);\theta_{i})\\ \varphi_{3}\left(\alpha ;\theta_{i}\right) & = & \psi ({\text{pr}}_{3}\left(\alpha \right);\theta_{i})\;, \end{array}$$

or shortly

$$\begin{array}{c} \varphi_{j}\left(\alpha ;\theta_{i}\right)=\psi \left({\text{pr}}_{j}\left(\alpha \right);\theta_{i}\right)=\cos^{c_{j}}\left(\theta_{i}-b_{j}\right) \end{array}$$ (28)

for *j* = 1, 2, 3 and *i* = 1,…,*m*. The matrix Φ(***α***) is the *m* × 3 matrix

$$\begin{array}{c} \Phi \left(\alpha \right)=\left[\begin{array}{ccc} \cos^{c_{1}}\left(\theta_{1}-b_{1}\right) & \cos^{c_{2}}\left(\theta_{1}-b_{2}\right) & \cos^{c_{3}}\left(\theta_{1}-b_{3}\right)\\ \cos^{c_{1}}\left(\theta_{2}-b_{1}\right) & \cos^{c_{2}}\left(\theta_{2}-b_{2}\right) & \cos^{c_{3}}\left(\theta_{2}-b_{3}\right)\\ \vdots & \vdots & \vdots \\ \cos^{c_{1}}\left(\theta_{m}-b_{1}\right) & \cos^{c_{2}}\left(\theta_{m}-b_{2}\right) & \cos^{c_{3}}\left(\theta_{m}-b_{3}\right) \end{array}\right]. \end{array}$$ (29)

For given ***α***, in C++ we calculate the matrices *U*, *V* and the vector of the singular values *s* by the command **svd**. The rank *r* of the matrix Φ(***α***) is equal to the number of nonzero singular values in *s*. We calculate the vector $\tilde{y}$ as in Eq (24) and solve the least square problems

$$\begin{array}{c} r_{2}\left(\alpha \right)=\left|\right|{\tilde{y}}_{\left[m-r\right]}\left(\alpha \right){\left|\right|}^{2}\;. \end{array}$$ (30)

As soon as the solution $\hat{\alpha }$ of the least square problem (30) is found we can calculate $\hat{a}$ (see Theorem 3) by $\hat{a}=\Phi^{+}\left(\hat{\alpha }\right)y$.

### The algorithms

In previous works [4, 6], the model described above was applied in conjunction with several custom-built algorithms that are based on local search heuristics and some meta-heuristics. The implemented algorithms include a steepest descen T algorithm, two iterative improvement algorithms with different neighbourhoods, and two genetic algorithms, a standard one and a hybrid one in which the best individuals of every generation are optimized with an iterative improvement algorithm. For a more detailed description of the algorithms we refer to [4]. The results of the experiments showed that all of the applied algorithms are capable of providing satisfactory results in all the tested instances, and differed mainly in the computational time needed. The average RMS values obtained on real lenses were around *RMS* = 2%. Hence, the above-mentioned results proved that the model is accurate, and that sufficiently good approximations can be found with a variety of algorithms for a sufficiently good description of lenses.

However, it should be remembered that the model can also be used for data compression tasks. A zero or very low RMS error is also essential in the foreseen application, in which pre-manufactured lenses need to be combined into a more complex luminaire with a prescribed light distribution.

In the model we use a sum of functions that are smooth, and hence the first and second derivatives can be calculated allowing application of continuous optimization methods in addition to the general discrete optimization meta-heuristics that were used before. We decided to use the Newton (also known as the Newton–Raphson) iterative method [11, 12] in order to find the sought-for solution. It is well known that convergence of the Newton method may significantly depend on the initial solution. We therefore applied the method in two ways. First, we used the Newton method as an optimizer which can pinpoint the local minimum of solutions found by heuristic algorithms. In a sense this implementation of the Newton method is an extension of the discrete optimization algorithm, which is used to finalize the search to end in a local minimum. (Note that the local minima may be missed by discrete optimization algorithms due to the predefined length of the discrete moves.) Secondly, we use the Newton method as a stand-alone algorithm that will, on initialization, generate a number of random (initial) solutions that are uniformly scattered over the whole search space. It then uses the Newton method on a number of the best initial solutions in order to find the local minima. Of course, for both implementations to be comparable, the iteration method has to be controlled so that the overall maximum amount of computation time is roughly the same.

#### Multi-start IF and IF-R

The **multi-start iterative improvement with fixed neighbourhood (IF) and IF with reduced parameters IF-R** algorithm [4–6] first initializes several initial solutions. The initial solutions are randomly chosen from the whole search space. Each of the initial solutions is then optimized using the following steps. At the beginning, the search step values (the step for numerical differentiation) *da* = 0.01, *db* = 1, and $dc=\frac{I_{max}}{10}$ are initialized, giving the 512 neighbours of the initial solution: (*a*_1_ ± *da*, *b*_1_ ± *db*, *c*_1_ ± *dc*, *a*_2_ ± *da*, *b*_2_ ± *db*, *c*_2_ ± *dc*, *a*_3_ ± *da*, *b*_3_ ± *db*, *c*_3_ ± *dc*). In the case of IF-R we only initialize the step values *db* = 1 and $dc=\frac{I_{max}}{10}$, giving 64 neighbours of the initial solution: (*b*_1_ ± *db*, *c*_1_ ± *dc*, *b*_2_ ± *db*, *c*_2_ ± *dc*, *b*_3_ ± *db*, *c*_3_ ± *dc*). The algorithm then randomly chooses a neighbour, and immediately moves to this neighbour if its RMS value is better than the current RMS value. If no better neighbour is found after 1000 trials, it is assumed that no better neighbour exists. In this case the algorithm morphs the neighbourhood by changing the step according to the formula *d*_*i*+1_ = *d*_*i*_ + *d*_0_. More precisely, *db*_*i*+1_ = *db*_*i*_ + *db*_0_ where *db*_0_ is the initial step value and by analogy *dc*_*i*+1_ = *dc*_*i*_ + *dc*_0_ where *dc*_0_ is the initial step value.

This is repeated until *i* = 10. If there still is no better solution, the initial step value is multiplied by 0.9 and the search is resumed from the current solution with a finer initial step. The algorithm stops when the number of generated solutions reaches *T*_*max*_.

#### Newton’s method IF-N

Newton’s method [11, 13, 14] is a well-known numerical optimization method, which can provide very good results under certain assumptions about the evaluation function and the initial solution. The evaluation function is indirectly minimized by looking for a solution of a system of nonlinear equations (the first derivatives of the evaluation function). Newton’s method solves the system of nonlinear equations iteratively by approximating it, in each step, with a system of linear equations which produce the delta vector. The delta vector is a part of the iterative scheme $x_{k}^{i+1}=x_{k}^{i}-d_{k}^{i}$. The Newton method converges when the delta vector vanishes, *d* = 0. At this point the evaluation coefficients found are the local minima. Details are given in [6]. An obvious assumption is that the evaluation function has to be a continuous non-linear function for which first and second order derivatives are defined. For Newton’s method to converge, the initial solution has to be close enough to a local or global optimum. For this reason the method is very sensitive to the choice of the initial solution.

### The datasets

**Fig 1:**
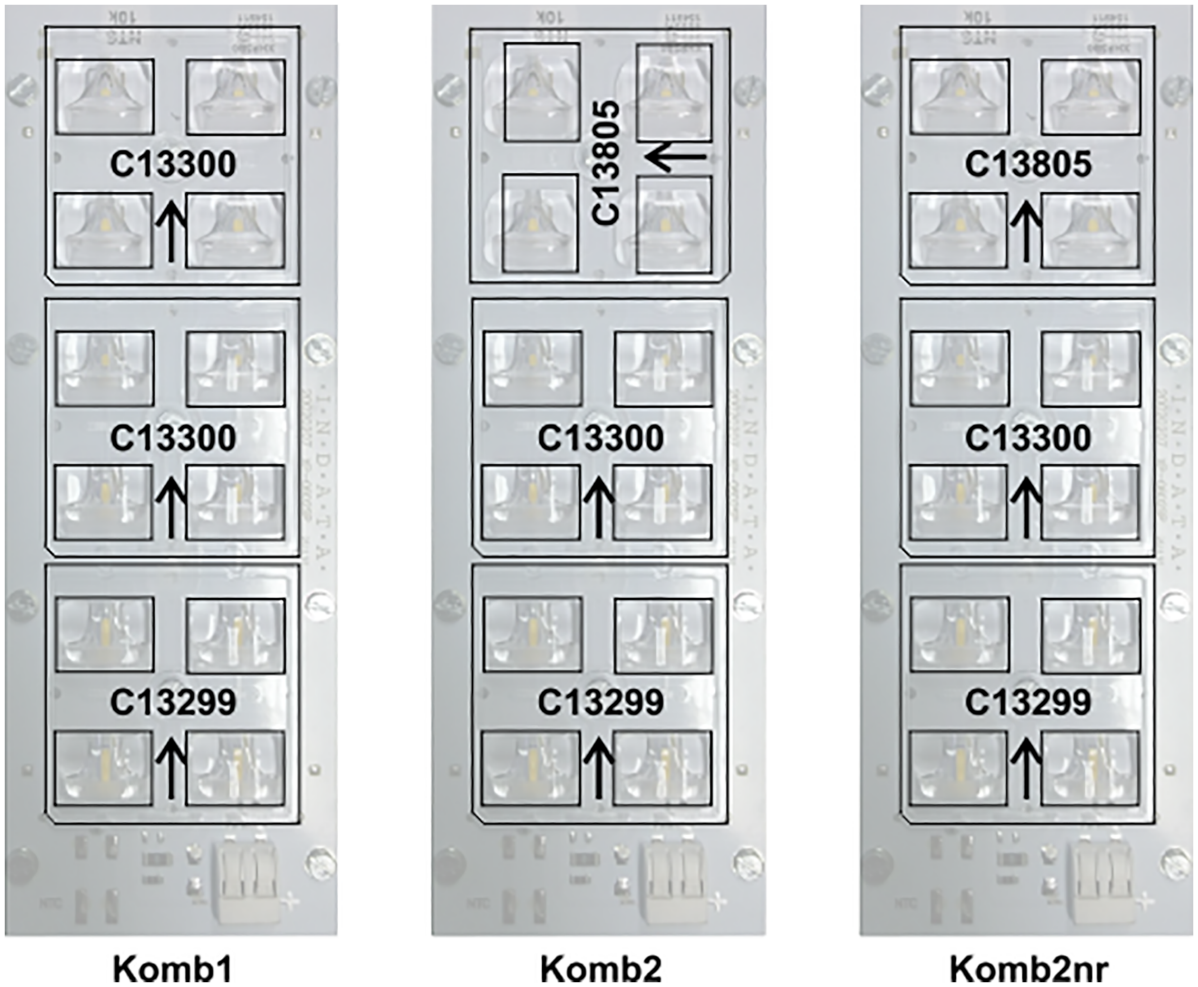
Graphical representation of the measured Ledil oy. lens combinations.

The experimental study made use of two batches consisting of 9 and 3 instances. We selected 9 different asymmetrical lenses which are meant to be used with a CREE XT-E series LED, from one of the world’s leading lens manufacturer LEDIL from Finland. We acquired the photometric data from LEDIL’s on-line catalogue [15]. The data was provided in.ies format, which we then converted to a vector list which is more suitable to use in our algorithms. LEDIL measured the individual lenses with a polar precision of 1° on 25 C panels. Additionally, we also ran the approximation on three LEDIL lenses that were measured in the photometric laboratory at the Faculty of Electrical Engineering in Ljubljana. These three (Komb1, Komb2 and Komb2nr) lenses from Fig 1 were measured at higher azimuth resolution, which yielded 13032 measurements and the same number of vectors to be approximated. The total number of real instances was 12.

## Experimental results and discussion

In this section the results obtained by using the three presented algorithms are discussed. All the algorithms ran for *T*_*max*_ = 4 million iterations. After every ten thousand iterations we saved the results which are presented in the section Supporting Information in S1, S2 and S3 Tables. The results are presented as the averages over 25 C-panels for each lens. The input data consisted of 12 lenses. The first 9 lenses were obtained from the manufacturers catalogue [15], and the last three were measured by us.

In S1, S2 and S3 Tables we present the average, minimum, and maximum RMS error after a varying number of iterations for the IF, IF-N and IF-R algorithms respectively. The types of lenses are given in the first columns. Experimentally, we also upgraded the IF algorithm with reduced parameters by the Newton method. Compared to S3 Table, the results of the IF-R algorithm upgraded by the Newton method are exactly the same. An obvious conclusion is that, at least in the cases tested, the results provided by the IF-R algorithm are so good that there is no room for improvement by the Newton method.

From the paper [6], we know that the IF-N algorithm improved the algorithms in [3, 4] by an average of 60% increased quality (minimized RMS). Here, S1 and S2 Tables show that the IF-N algorithm improves the classical algorithm IF slightly. However, we are more interested in a comparison of the results of the RMS errors of the IF-R algorithm with respect to the data of the IF algorithm (or the IF-N algorithm).

We can immediately see that our new algorithm (IF-R) is, on average, much better than both the IF and the IF-N algorithms. The errors after 4M iterations are much lower in our new algorithm with respect to the usual IF algorithm. On the other hand, the errors after 4M iterations of the new algorithm and the IF-N algorithm are comparable.

In order to analyze the results given in S1, S2 and S3 Tables more closely let us consider the graphs provided below. On the horizontal axis we have the number of iterations, and on the vertical axis the RMS error. The average convergence curves for the whole lenses are very similar, so that are not presented here. Instead we chose one lens, the Komb1 (all 25 C-planes) to present the results and comparisons, so that we can see the characteristic differences between the C-plane approximations.

**Fig 2:**
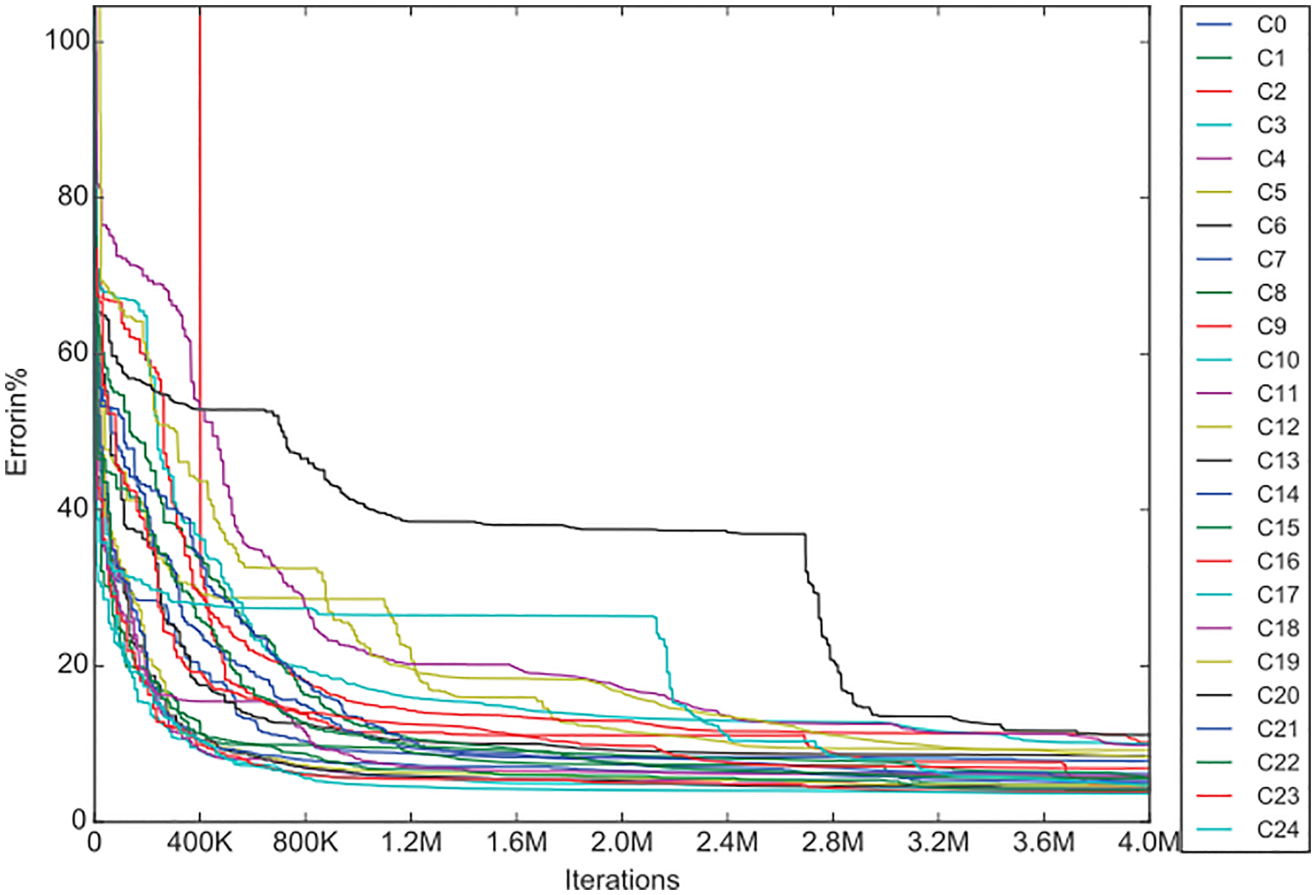
Convergence curves of the lens Komb1 for the *C*-planes of the IF algorithm. The curves in the graph are the best on each C-panel regardless of the multi-start. In other words C0 can be obtained from multi-start 5 and C1 from multi-start 9. The criteria for the best approximation is the RMS obtained at *T*_*max*_.

In Fig 2, the convergence curves of Komb1 from the IF algorithm are given for each *C*-plane. It can be seen that the improvement of the errors seems to be insignificant between 3.2M and 4M iterations. It can also be observed that, while the majority of the C-planes follow the same convergence pattern, there are some that deviate. We could attribute this deviation to poorly chosen initial solutions, or to specific input data which can cause the search algorithms to struggle (see C17 and C20 in Fig 2). As the experiment was set up in multi-start mode, which means that every approximation started 10 times with randomly chosen initial solutions, and that the presented data is the best of the best it is more likely that the convergence curve deviation is due to specific input data. However it can be seen that, in the end, no matter which input was used and how the convergence curve looks like, all the RMS at *T*_*max*_ are similar.

**Fig 3:**
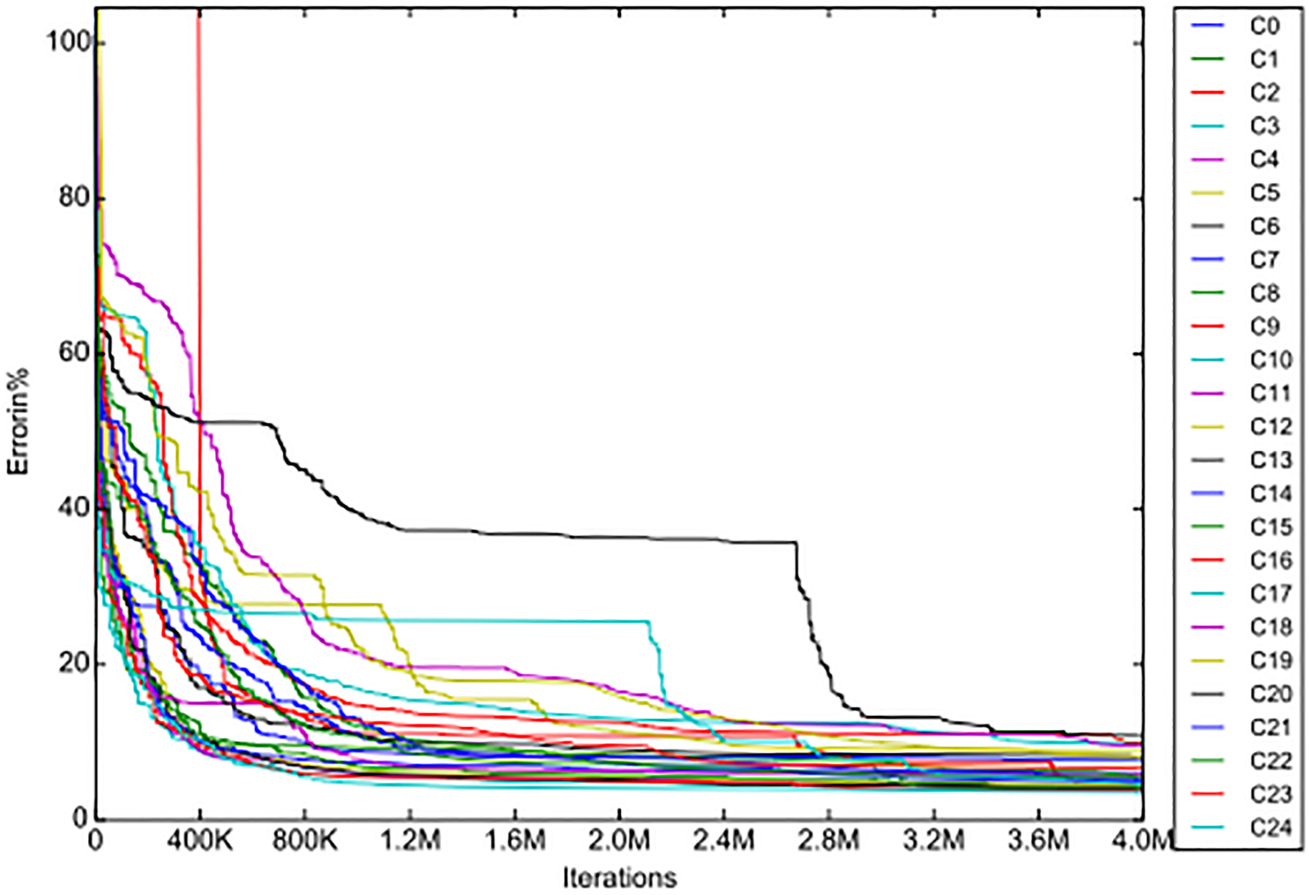
The convergence of the IF-N algorithm.

**Fig 4:**
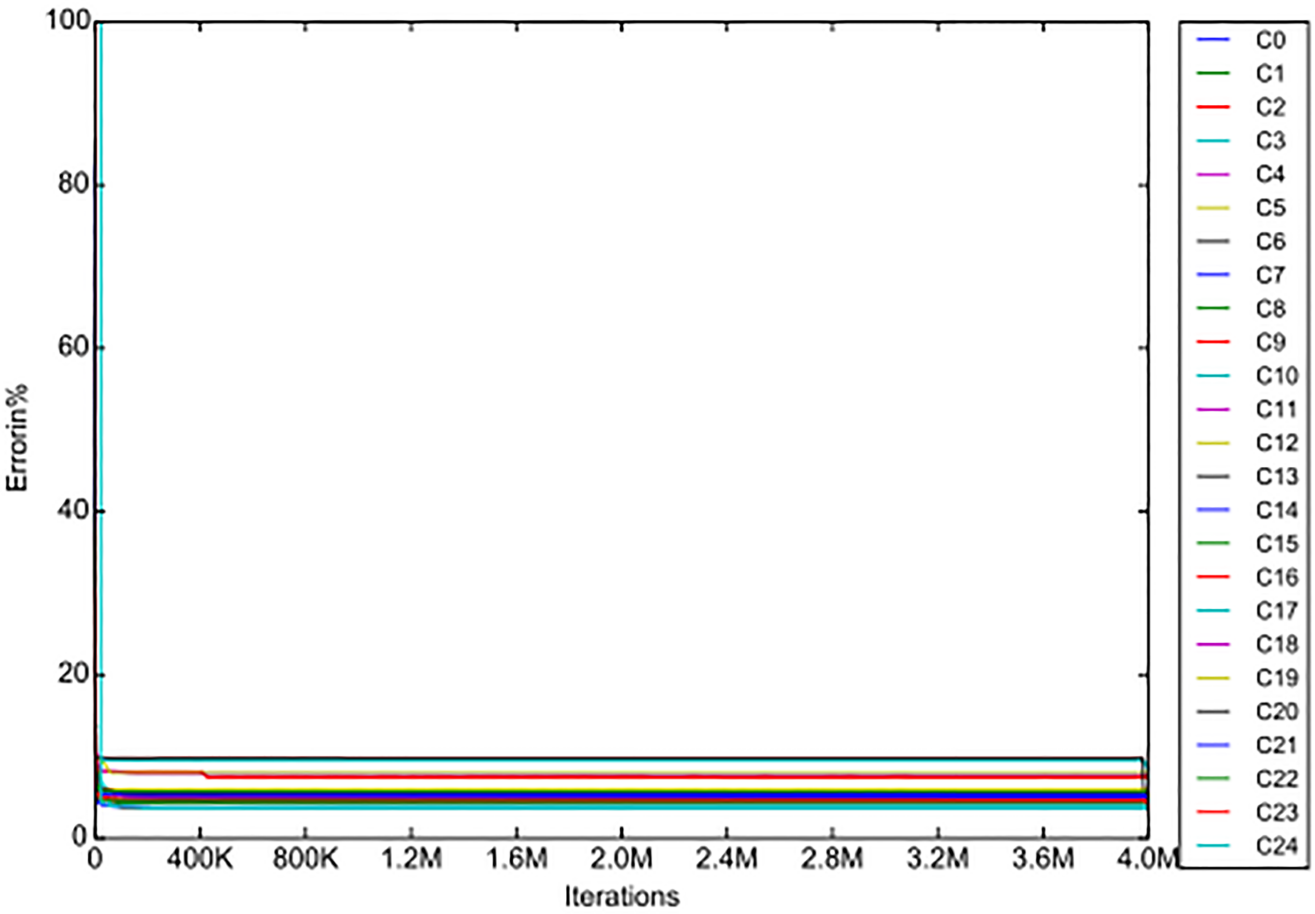
The convergence of the IF-R algorithm.

A comparison of the convergence of the IF-N algorithm and of the IF-R algorithm for the Komb1 lens is represented in the next figures (Figs 3, 4), where in both graphs the same scale is used.

**Fig 5:**
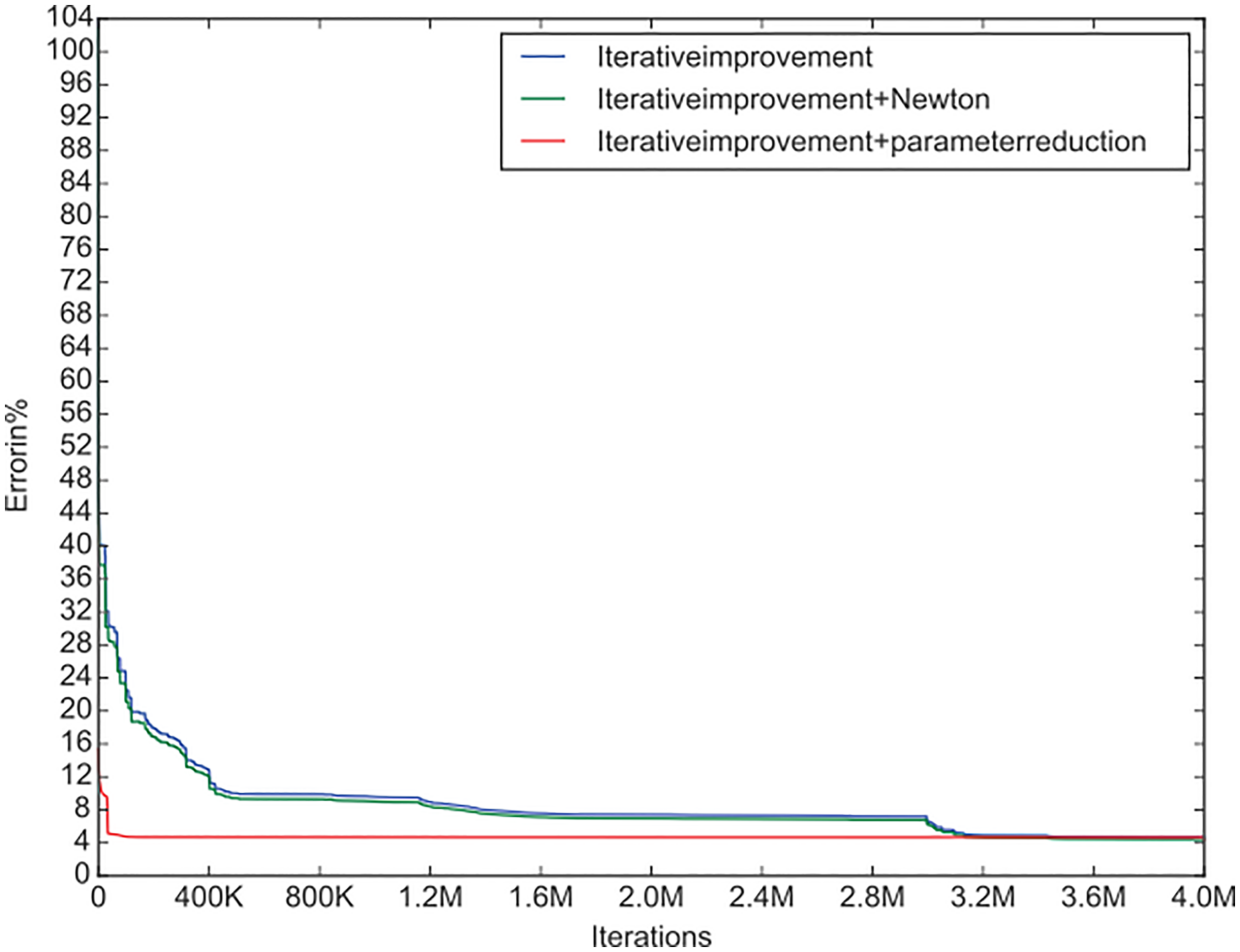
Convergence curves of the Komb1 lens for the *C*-plane 1.

We chose the IF-N algorithm because it exhibits almost the same convergence rate as the IF algorithm, but provides slightly better end results (see Fig 5). This makes it more appropriate for comparisons with the new algorithm.

**Fig 6:**
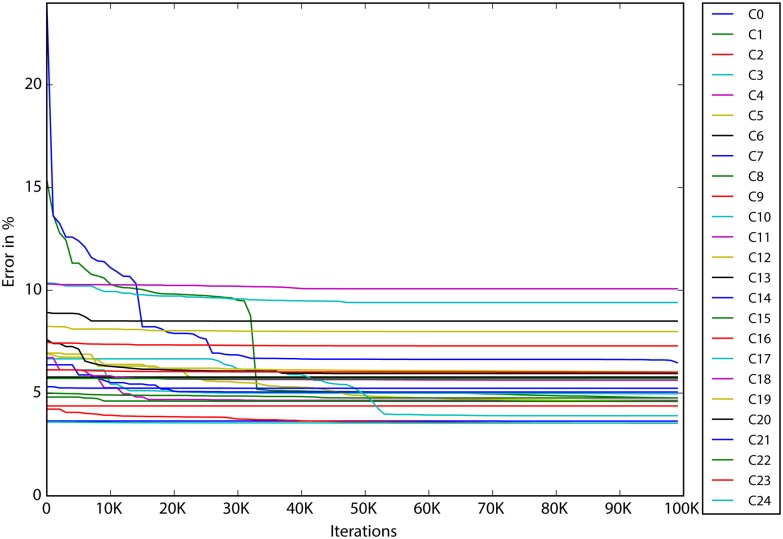
Convergence of the IF-R algorithm with reduced parameters in 100K iterations.

Clearly, the convergence of the IF-R algorithm is much faster than that of the IF-N algorithm. In fact it is so fast that the scale used does not illustrate any properties of the new algorithm’s convergence apart from its speed. For this reason we looked at the graph in Fig 4 on a smaller horizontal scale and got the following graph (Fig 6)

The convergence of the IF-R algorithm is 40 times faster than that of the IF and IF-N algorithms, but the curve slopes (gradients) are still similar, just on a smaller scale. Also, similarly to the previous algorithms the IF-R algorithm shows only minute errors after 100K iterations. From this it can be concluded that parameter separation has a huge impact on computation time but does not alter the algorithm behaviour when searching for the best solution, which is somewhat expected, as we did not change the algorithm workflow, just the pool of possible solutions.

Next we take out the graphs for the *C*1 plane from the figures above, and from the data of the iterations we add the convergence curve of the IF-N algorithm. The convergence curves of all three algorithms (IF, IF-N and IF-R) for the C1 plane of the Komb1 lens are displayed in Fig 5. From this it can be seen that the IF-N algorithm does not change the convergence curve as was implied above, it just improves the RMS values slightly at every step. We again observe the superiority of the algorithm with reduced parameters, which converges in a fraction of the time needed for the other two algorithms.

**Fig 7:**
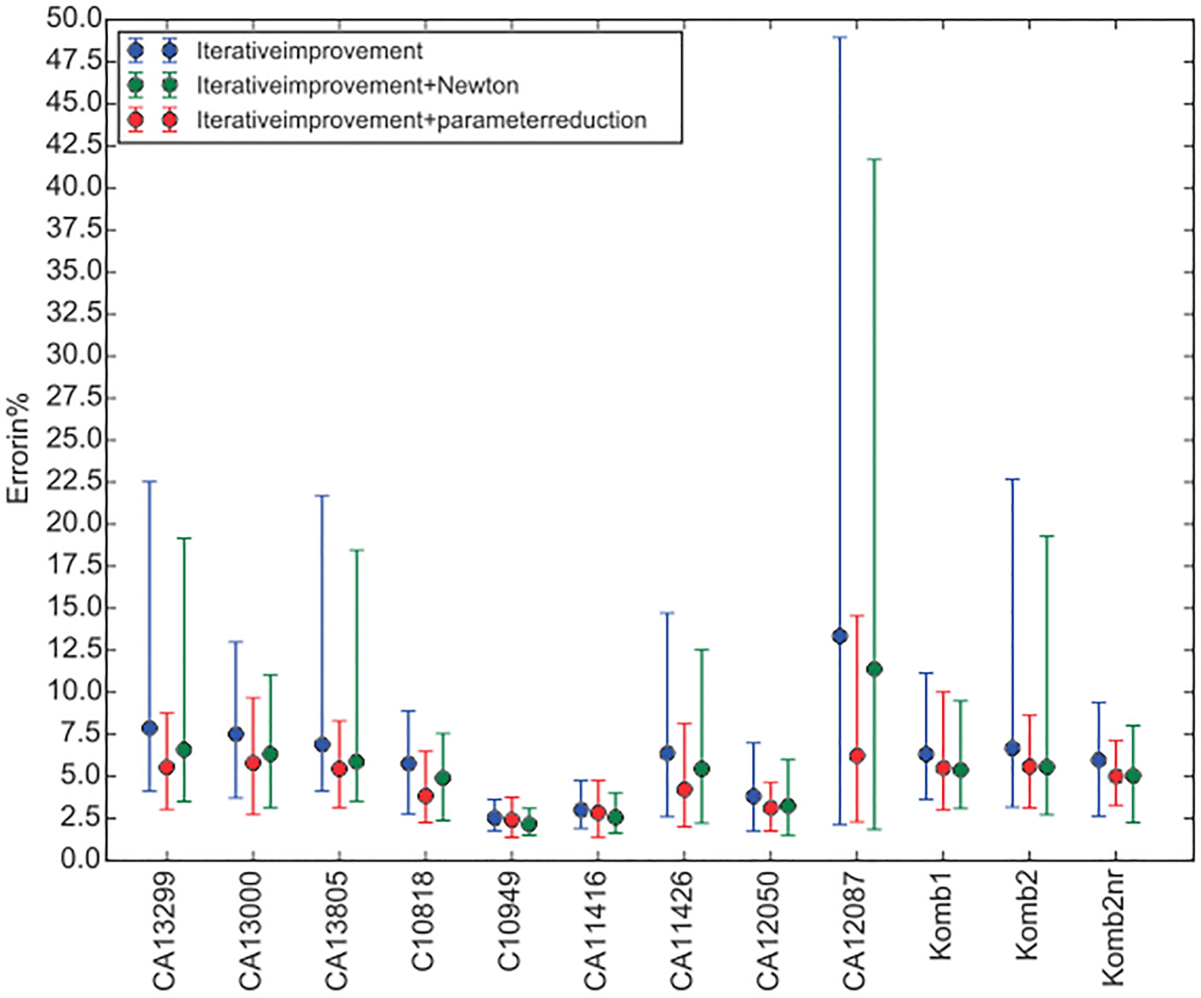
Min-Avg-Max scatter diagram.

For the last comparison of the algorithms, we showcase a scatter diagram of all the lenses in Fig 7. This diagram shows the RMS values at *T*_*max*_ for the best, the worst, and the average over 25 C-panels of each lens from the experiment.

Again there is an obvious superiority of the new algorithm drawn in red, where the differences between the maximum (worst) RMS and the minimum (best) RMS are the smallest. It can be seen that the differences in the case of the new algorithm are mostly two times smaller than in the case of the old algorithms, but they can also be 4 times smaller as in case of the CA12087 lens.

It can therefore be concluded that the used method of separating the linear and non-linear parameters, provides a huge performance boost to the developed algorithms, even to the level where these algorithms could be used in commercial applications, which are meant to be used on a daily basis by users who are not programmers or mathematicians.

**Remark 4** *Following the recommendation of one of the reviewers, our results were compared with the results of the standard optimization solver in Matlab. The standard function* **lsqcurvefit** *for approximation of nonlinear function given by* Eq (1) *was used*. *For the initial values, random solutions (i.e. vectors from* [0, 1]^3^ × [−90, 90]^3^ × [0, 100]^3^*) were generated, and the measured data were the measured luminous values I*_*M*_(*θ*_*i*_) at polar angles *θ*_*i*_ *(see section The model)*. *We ran* **lsqcurvefit** *for all 25 C-panels for each of the first 9 lenses*. *To allow approximately the same running time (Matlab was run on another computer), we aim to allow approximately the same number of feasible solutions generated by each of the solvers. Therefore, on each instance (each C panel of each lens), function* **lsqcurvefit** *was restarted 50 times with 40 000 iterations allowed and precision 1e-6*. *The best solutions on each instance are memorised*. *In* S4 Table, *the RMS error on best, worst and average C panel are reported (right rows)*. *Roughly speaking, comparison with the average, minimum, and maximum RMS error obtained IF-R algorithm (left rows) shows that in most cases, the solutions are of similar quality*. *More precisely, on lens CA12087, Matlab solver provides solutions with significantly lower RMS error (two to three times better), but on the other hand, on lenses CA13299, CA13300 and CA13805, the IF-R solutions are about 6 times better on average! Note that on these three lenses, the Matlab solver was in addition run with 500 restarts, with 400 000 iterations allowed and with precision 1e-10, and in all cases, the solutions did not improve significantly*. *On the other hand, note that IF-R finds solutions of the reported quality in much shorter runs (see* Figs 3 and 4 *), and was allowed 4M iterations just to show the speed up in comparison to the other algorithms tested. This additional experiment thus showed that the reduction of parameters dramatically improves the convergence speed, and sometimes also the quality of approximation*. *An interesting avenue of further research may be to optimize the implementation and parameter tuning of IF-R, and, on the other hand, understand the properties of the instances (c.f. CA13299, CA13300 and CA13805) that allowed so diverse solution qualities*.

## Supporting information

S1 Table RMS error values for IF. The Average sub-table presents the average data over 25 C-panels, the Min table the best, and the Max table the worst C-panel.
(PDF)

S2 Table RMS error values for IF-N. The Average sub-table presents the average data over 25 C-panels, the Min table the best, and the Max table the worst C-panel.
(PDF)

S3 Table RMS error values for IF-R. The Average sub-table presents the average data over 25 C-panels, the Min table the best, and the Max table the worst C-panel.
(PDF)

S4 Table RMS error values for IF-R and Matlab. The average, the best and the worst RMS error of IF-R AND Matlab solver after around 4M iterations.
(PDF)
