# Supplementary material for: Improved approximation of spatial light distribution
Source: PLoS One. 2017 Apr 28;12(4):e0176252. doi: 10.1371/journal.pone.0176252 (PMC5409530; doi:10.1371/journal.pone.0176252)
Supplement: S1 Table — The Average sub-table presents the average data over 25 C-panels, the Min table the best, and the Max table the worst C-panel. (PDF) [file pone.0176252.s001.pdf]

S1 Table. RMS error values for IF. The Average sub-table presents the average data over 25 C-panels, the Min table the best, and the Max table the worst C-panel.

Average									
L \ I	10K	20K	40K	100K	200K	500K	1M	2M	4M
CA13299	18,4796	18,4796	18,4796	18,3781	17,1909	16,0362	8,9394	8,0405	7,8750
CA13300	20,9503	20,9503	20,9503	20,8675	20,1026	18,8059	9,6723	7,7704	7,5206
CA13805	17,4319	17,4319	17,4319	17,4319	15,5339	13,1424	7,3928	6,9644	6,9049
C10818	21,9478	21,9478	21,9478	21,7269	21,1157	20,6192	9,1653	6,4107	5,7647
C10949	9,8835	9,8835	9,8335	9,7984	7,9491	7,1432	3,1941	2,6262	2,5407
CA11416	12,7859	12,7859	12,6445	12,5752	10,2956	8,7888	3,8203	3,1195	3,0150
CA11426	23,3820	23,3820	23,3820	23,3820	23,0741	20,2419	8,4431	6,7436	6,3816
CA12050	14,2652	14,2652	14,2652	14,2652	12,0097	10,1818	5,0558	4,0226	3,8284
CA12087	31,2364	31,2364	31,2364	30,9354	29,0792	28,5316	18,2022	14,5474	13,3465
Komb1	25,9090	25,9090	25,8245	25,8245	22,6201	17,9048	7,7915	6,5852	6,3243
Komb2	17,5343	17,5343	17,5343	17,5343	15,9877	13,0758	7,3554	6,7823	6,6767
Komb2nr	17,7418	17,7418	17,7418	17,7014	15,8705	14,0878	6,7797	6,1923	5,9813

Min									
L \ I	10K	20K	40K	100K	200K	500K	1M	2M	4M
CA13299	4,1484	4,1484	4,1484	4,1484	4,1484	4,1484	4,1484	4,1484	4,1210
CA13300	5,4079	5,4079	5,4079	5,4079	5,4079	5,4079	4,0451	3,7269	3,7136
CA13805	6,5678	6,5678	6,5678	6,5678	6,5678	6,5678	4,3384	4,1514	4,1514
C10818	4,6791	4,6791	4,6791	4,6791	4,6791	4,6791	4,4907	3,7675	2,7815
C10949	2,2246	2,2246	2,2246	2,2246	2,2246	2,2246	1,9037	1,7745	1,7723
CA11416	2,1694	2,1694	2,1694	2,1694	2,1694	2,1694	2,1694	1,9319	1,9247
CA11426	4,1764	4,1764	4,1764	4,1764	4,1764	4,1764	2,8835	2,6316	2,5979
CA12050	2,5320	2,5320	2,5320	2,5320	2,5320	2,5320	1,9549	1,7685	1,7376
CA12087	3,0794	3,0794	3,0794	3,0794	3,0794	3,0794	3,0794	2,3914	2,1570
Komb1	4,5552	4,5552	4,5552	4,5552	4,5552	4,5552	3,9091	3,6438	3,6271
Komb2	3,8535	3,8535	3,8535	3,8535	3,8535	3,8535	3,7953	3,2355	3,1879
Komb2nr	3,7762	3,7762	3,7762	3,7762	3,7762	3,7762	3,7398	2,7597	2,6464

Max									
L \ I	10K	20K	40K	100K	200K	500K	1M	2M	4M
CA13299	49,2730	49,2730	49,2730	49,2730	47,7399	43,7265	22,5463	22,5264	22,5234
CA13300	65,2438	65,2438	65,2438	65,2438	65,2438	50,7972	28,2060	13,5235	12,9900
CA13805	29,6394	29,6394	29,6394	29,6394	29,5346	29,5346	21,9813	21,7168	21,7155
C10818	47,1243	47,1243	47,1243	47,1243	47,1243	47,1243	19,7952	9,7906	8,8865
C10949	17,5955	17,5955	17,5955	17,5955	13,8012	12,5645	5,1902	3,9234	3,6391
CA11416	43,4536	43,4536	43,4536	41,7225	29,9575	28,5790	7,6618	4,8232	4,7527
CA11426	90,5431	90,5431	90,5431	90,5431	90,5431	64,4384	16,2986	14,7818	14,7243
CA12050	50,6776	50,6776	50,6776	50,6776	39,9701	30,1798	9,8187	7,2841	6,9919
CA12087	84,3584	84,3584	84,3584	84,3584	84,3584	84,3584	76,9588	57,7387	48,9436
Komb1	68,9868	68,9868	66,8761	66,8761	66,2175	45,1308	16,7848	11,3910	11,1609
Komb2	42,1196	42,1196	42,1196	42,1196	38,2335	26,4035	22,7677	22,6761	22,6737
Komb2nr	45,8413	45,8413	45,8413	45,8413	44,8202	44,8202	11,5014	10,0511	9,4118
