# Supplementary material for: Improved approximation of spatial light distribution
Source: PLoS One. 2017 Apr 28;12(4):e0176252. doi: 10.1371/journal.pone.0176252 (PMC5409530; doi:10.1371/journal.pone.0176252)
Supplement: S2 Table — The Average sub-table presents the average data over 25 C-panels, the Min table the best, and the Max table the worst C-panel. (PDF) [file pone.0176252.s002.pdf]

S2 Table. RMS error values for IF-N. The Average sub-table presents the average data over 25 C-panels, the Min table the best, and the Max table the worst C-panel.

L \ I	10K	20K	40K	100K	200K	500K	1M	2M	4M
**Average**									
CA13299	16,8671	16,8671	16,3575	15,9681	14,6454	13,8321	7,5141	6,7694	6,5958
CA13300	19,3416	19,3416	18,4668	18,0027	17,3061	16,1753	8,3499	6,5930	6,3370
CA13805	16,2189	16,2189	15,3367	15,0447	13,3652	11,3389	6,3691	5,9465	5,8799
C10818	20,6125	20,6125	19,6377	18,8161	18,1175	17,6458	7,7672	5,5342	4,9167
C10949	9,1898	9,1898	8,6425	8,4342	6,8397	6,1427	2,7683	2,2613	2,1655
CA11416	11,2791	11,2791	10,6827	10,3811	8,6604	7,3709	3,3077	2,6808	2,5716
CA11426	21,6286	21,6286	20,9017	20,2541	19,7784	17,4293	7,2842	5,8217	5,4516
CA12050	12,9019	12,9019	12,5059	12,2332	10,3242	8,7550	4,3567	3,4471	3,2497
CA12087	29,1777	29,1777	28,0626	26,8131	25,0508	24,5457	15,6448	12,4867	11,3855
Komb1	23,7304	23,7304	22,8628	22,2562	19,4869	15,4079	6,6889	5,6062	5,3804
Komb2	15,0802	15,0802	14,3796	13,9969	12,8902	10,8923	6,3193	5,6681	5,5633
Komb2nr	15,7815	15,7815	15,2013	14,9272	13,4432	12,1254	5,7668	5,2716	5,0484
**Min**									
CA13299	3,7940	3,7940	3,5413	3,5413	3,5413	3,5413	3,5413	3,5268	3,5067
CA13300	4,7136	4,7136	4,6567	4,6567	4,6567	4,6567	3,4709	3,1933	3,1603
CA13805	6,3833	6,3833	5,6630	5,6630	5,6343	5,6343	3,7305	3,5315	3,5315
C10818	4,3596	4,3596	4,1227	4,1052	4,0734	4,0734	3,8547	3,2250	2,3825
C10949	2,1633	2,1633	1,9450	1,8969	1,8952	1,8926	1,6265	1,5232	1,5076
CA11416	2,0937	2,0937	2,0849	1,9032	1,9032	1,8575	1,8470	1,6553	1,6374
CA11426	3,6630	3,6630	3,6548	3,6478	3,5671	3,5671	2,5110	2,2401	2,2120
CA12050	2,2518	2,2518	2,2518	2,2098	2,2098	2,2039	1,7092	1,5081	1,4792
CA12087	2,7335	2,7335	2,7335	2,6296	2,6296	2,6296	2,6296	2,0354	1,8373
Komb1	4,2483	4,2483	4,1385	3,9089	3,9089	3,9089	3,3415	3,1164	3,0926
Komb2	3,8018	3,8018	3,3936	3,3912	3,3033	3,3033	3,2327	2,7586	2,7136
Komb2nr	3,7119	3,7119	3,2657	3,2441	3,2441	3,2441	3,2285	2,3803	2,2509
**Max**									
CA13299	46,4490	46,4490	44,9290	43,4382	42,1442	39,0822	19,3052	19,1652	19,1652
CA13300	59,4229	59,4229	59,4229	55,8746	55,6304	44,3638	24,6804	11,6910	11,0515
CA13805	28,8678	28,8678	27,2566	25,6208	25,1310	25,1310	18,7574	18,5006	18,4720
C10818	45,9907	45,9907	42,3925	40,8124	40,4855	40,4855	16,9266	8,5248	7,5739
C10949	16,6701	16,6701	15,3195	15,0617	11,9820	10,7393	4,4815	3,3729	3,1045
CA11416	34,4724	34,4724	33,1057	30,5552	25,5852	24,4474	6,8312	4,1498	4,0423
CA11426	83,8017	83,8017	83,8017	78,4189	77,5024	56,3666	13,9815	12,7940	12,5564
CA12050	45,3377	45,3377	44,7164	43,7072	34,6360	25,9573	8,4274	6,2391	5,9927
CA12087	79,4149	79,4149	79,4149	73,6744	73,6744	73,6744	65,6855	49,3370	41,6841
Komb1	64,2557	64,2557	59,7255	59,1171	56,8743	38,6108	14,7529	9,6998	9,4881
Komb2	35,9869	35,9869	34,6141	32,6112	32,6112	22,7049	19,4098	19,3436	19,2837
Komb2nr	41,7039	41,7039	41,1877	41,1466	38,2767	38,2767	10,1278	9,0415	8,0208
