# Supplementary material for: Improved approximation of spatial light distribution
Source: PLoS One. 2017 Apr 28;12(4):e0176252. doi: 10.1371/journal.pone.0176252 (PMC5409530; doi:10.1371/journal.pone.0176252)
Supplement: S3 Table — The Average sub-table presents the average data over 25 C-panels, the Min table the best, and the Max table the worst C-panel. (PDF) [file pone.0176252.s003.pdf]

S3 Table. RMS error values for IF-R. The Average sub-table presents the average data over 25 C-panels, the Min table the best, and the Max table the worst C-panel.

Average									
L \ I	10K	20K	40K	100K	200K	500K	1M	2M	4M
CA13299	6,0419	5,7481	5,6141	5,5630	5,5582	5,5558	5,5534	5,5505	5,5469
CA13300	6,7611	6,2789	6,0280	5,8451	5,8226	5,8152	5,8123	5,8094	5,8047
CA13805	6,2912	5,6959	5,5680	5,5249	5,5201	5,5091	5,5032	5,4549	5,4515
C10818	5,6187	4,8123	4,2481	3,8865	3,8747	3,8640	3,8601	3,8546	3,8481
C10949	2,9175	2,6776	2,4880	2,4285	2,4246	2,4233	2,4221	2,4205	2,4189
CA11416	3,4155	3,2505	3,1065	2,9604	2,9055	2,8758	2,8621	2,8522	2,8460
CA11426	5,7680	5,0397	4,4958	4,2501	4,2268	4,2255	4,2246	4,2238	4,2228
CA12050	3,8529	3,6624	3,5169	3,2644	3,2219	3,1919	3,1611	3,1442	3,1278
CA12087	8,4827	7,6333	6,6182	6,4527	6,4243	6,3347	6,2962	6,2630	6,2402
Komb1	6,1349	5,8181	5,6765	5,5716	5,5579	5,5429	5,5352	5,5112	5,5049
Komb2	6,5542	6,1175	5,8482	5,6961	5,6870	5,6814	5,6757	5,6050	5,5943
Komb2nr	5,7709	5,3995	5,2608	5,1478	5,1108	5,0746	5,0406	5,0152	5,0107
Min									
L \ I	10K	20K	40K	100K	200K	500K	1M	2M	4M
CA13299	3,4019	3,1483	3,0665	3,0559	3,0552	3,0542	3,0531	3,0523	3,0511
CA13300	2,7403	2,7403	2,7316	2,7301	2,7295	2,7285	2,7271	2,7256	2,7243
CA13805	4,0289	3,9700	3,3843	3,1782	3,1687	3,1632	3,1596	3,1585	3,1574
C10818	2,6139	2,6139	2,4967	2,2657	2,2656	2,2657	2,2657	2,2657	2,2657
C10949	2,1162	1,6323	1,4068	1,3903	1,3885	1,3872	1,3862	1,3855	1,3841
CA11416	2,5683	2,3365	1,7614	1,4114	1,4030	1,4029	1,4029	1,4029	1,4029
CA11426	3,4572	2,8399	2,2568	2,0309	2,0174	2,0173	2,0173	2,0171	2,0170
CA12050	2,1984	2,1080	1,9706	1,7507	1,7507	1,7507	1,7507	1,7507	1,7507
CA12087	3,2171	2,8671	2,7567	2,5348	2,5094	2,3094	2,3093	2,3093	2,3093
Komb1	3,6445	3,6445	3,4473	3,0336	3,0334	3,0331	3,0328	3,0324	3,0317
Komb2	3,3822	3,1707	3,1246	3,1223	3,1221	3,1221	3,1220	3,1218	3,1213
Komb2nr	3,3978	3,3978	3,2981	3,2943	3,2941	3,2939	3,2938	3,2937	3,2933
Max									
L \ I	10K	20K	40K	100K	200K	500K	1M	2M	4M
CA13299	9,1497	8,9455	8,8311	8,7987	8,7986	8,7985	8,7985	8,7985	8,7985
CA13300	12,6862	12,1551	9,9985	9,7894	9,7338	9,7131	9,7012	9,6994	9,6981
CA13805	11,5491	8,3821	8,3695	8,3367	8,3346	8,3320	8,3305	8,3264	8,3238
C10818	10,2955	9,0103	6,5415	6,4917	6,4907	6,4907	6,4907	6,4907	6,4907
C10949	4,4072	3,7678	3,7678	3,7678	3,7678	3,7678	3,7678	3,7678	3,7678
CA11416	4,8057	4,7984	4,7916	4,7806	4,7805	4,7805	4,7805	4,7805	4,7805
CA11426	10,7993	10,1067	8,9240	8,1726	8,1723	8,1723	8,1723	8,1723	8,1723
CA12050	5,3383	5,2617	5,0653	4,9932	4,8990	4,7768	4,6930	4,6720	4,6720
CA12087	18,8342	17,4985	14,7553	14,5809	14,5809	14,5809	14,5809	14,5809	14,5809
Komb1	10,0683	10,0683	10,0601	10,0585	10,0562	10,0552	10,0551	10,0544	10,0543
Komb2	9,6962	9,1915	9,1889	8,6904	8,6735	8,6730	8,6730	8,6730	8,6730
Komb2nr	7,7887	7,2572	7,2267	7,1254	7,1251	7,1251	7,1251	7,1251	7,1251
