# Supplementary material for: Improved approximation of spatial light distribution
Source: PLoS One. 2017 Apr 28;12(4):e0176252. doi: 10.1371/journal.pone.0176252 (PMC5409530; doi:10.1371/journal.pone.0176252)
Supplement: S4 Table — The average, the best and the worst RMS error of IF-R AND Matlab solver after around 4M iterations. (PDF) [file pone.0176252.s004.pdf]

S4 Table. RMS error values for IF-R and Matlab. The average, the best and the worst RMS error of IF-R AND Matlab solver after around 4M iterations.

	IF-R			Matlab		
Lenses	Av	Min	Max	Av	Min	Max
CA13299	5,5469	3,0511	8,7985	33,2667	15,0128	55,9850
CA13300	5,8047	2,7243	9,6981	33,4248	14,8933	72,1478
CA13805	5,4515	3,1574	8,3238	30,6230	15,6739	52,0547
C10818	3,8481	2,2657	6,4907	3,8723	1,0480	11,1625
C10949	2,4189	1,3841	3,7678	2,9832	1,1202	5,9739
CA11416	2,8460	1,4029	4,7805	3,0106	1,5702	4,7451
CA11426	4,2228	2,0170	8,1723	2,8565	1,2021	5,6571
CA12050	3,1278	1,7507	4,6720	2,8901	1,4853	5,2273
CA12087	6,2402	2,3093	14,5809	2,4360	0,6964	4,1419
